# Evaluation of a Multidimensional Occupational Therapy Environmental Checklist for People Experiencing Delirium During Hospital Admission: A Quasi‐Experimental Study

**DOI:** 10.1111/ajag.70165

**Published:** 2026-04-24

**Authors:** Sharon Neale, Erin McKnight, Renee Dixon, Danielle Hitch

**Affiliations:** ^1^ Occupational Therapy Western Health Melbourne Victoria Australia; ^2^ Occupational Science and Therapy, School of Health and Social Development Deakin University Geelong Victoria Australia

**Keywords:** aged, delirium, functional status, health facility environment, inpatients, occupational therapy

## Abstract

**Objective:**

Delirium, an acute medical emergency, significantly impacts older adults, increasing morbidity, mortality and healthcare costs (estimated at $8.8 billion annually in Australia). Environmental modifications in hospital wards are underexplored despite their potential to mitigate delirium's effects. This study evaluated a multidimensional occupational therapy environmental checklist's impact on functional and service outcomes for hospitalised delirium patients compared to standard care.

**Methods:**

A quasi‐experimental design was employed, collecting pre‐ and post‐intervention data from 100 electronic medical records (50 comparison, 50 intervention) on a Geriatric Evaluation and Management ward in Melbourne, Australia. The checklist, implemented by occupational therapists and allied health assistants, targeted orientation, object accessibility, daily routines and safety. Outcomes included length of stay, adverse events (e.g., falls, pressure injuries) and Functional Independence Measure (FIM) scores. Descriptive statistics, *t*‐tests and *χ* ^2^ tests were conducted using SPSS Statistics 28 (*p* < 0.05).

**Results:**

The intervention group showed a 27% reduction in total adverse events (comparison: *n* = 37; intervention: *n* = 27) and significantly higher FIM scores at discharge (motor: *t* = −2.38, *p* = 0.02; cognitive: *t* = −2.62, *p* = 0.01; total: *t* = −3.24, *p* < 0.001). However, length of stay (comparison: M = 28.2 days; intervention: M = 29.36 days; *t* = −0.20, *p* = 0.84) and adverse event rates (*χ* ^2^ = 1.48, *p* = 0.22) did not differ significantly. The intervention group had a higher fall admission rate (36% vs. 2%; *χ* ^2^ = 20.38, *p* < 0.001).

**Conclusions:**

The checklist enhances functional recovery in older adults with delirium, reducing adverse events. Larger, multi‐site studies are needed to confirm efficacy and generalisability, supporting occupational therapy's role in delirium management.

## Abstract (highlights)

**Policy Impact:**

The findings of this study support the key role and contributions of occupational therapy in delirium management. Policymakers could consider funding multi‐site trials and staff education to standardise this low‐cost intervention, improving care quality for ageing populations.

**Practice Impact:**

The environmental checklist enhances delirium care by improving functional outcomes in older adults. Occupational therapists and multidisciplinary teams can adopt this practical tool, supported by training and family involvement, to optimise hospital environments and patient recovery.

## 1 Introduction

Delirium is an acute medical emergency characterised by disturbed consciousness, cognition, attention and perception, with rapid onset and fluctuating presentation [1]. It is linked to increased institutionalisation, morbidity, mortality and substantial healthcare costs [2]. In Australia, the total annual cost of delirium has been estimated at $A8.8 billion, highlighting its substantial national impact [3, 4]. Individuals with delirium face elevated risks of pressure injuries, dementia, falls and extended hospital stays [5].

Effective delirium management requires a coordinated response from healthcare professionals, including nurses, doctors and allied health staff (ACSQHC, 2021). Evidence supports multicomponent interventions targeting risk factors such as immobility, disorientation and sensory deficits, with strategies such as early mobilisation, re‐orientation and reduced psychoactive drug use proving effective [6]. However, interventions explicitly addressing hospital ward environments are less common, despite their potential to mitigate delirium's impact. Mudge et al. [5] implemented a multidisciplinary approach with occupational therapists and physiotherapists, introducing delirium bays, orientation boards and appropriate lighting, resulting in fewer patients discharged with persistent delirium (32% vs. 71%; *p* = 0.02).

In a single‐blind randomised controlled trial of 287 older medical inpatients at intermediate or high risk of delirium, Martinez et al. [7] found that a family delivered multicomponent environmental intervention reduced delirium incidence from 13% in the control group to 6% in the intervention group (relative risk 0.41, 95% CI 0.19–0.92; *p* = 0.03). Chong et al. [8, 9, 10] demonstrated the benefits of bright light therapy specifically, improving sleep and function across a series of cohort studies. Across these studies, multicomponent environmental strategies were associated with clinically meaningful reductions in delirium incidence or persistence, supporting a focus on ward level modification.

Occupational therapy (OT) has professional expertise in environmental modification, and the role of occupational therapists has been highlighted in recent studies [11, 12, 13]. Sheard et al. [11] reported a 25% reduction in delirium incidence with personalised orientation boards, while Harper et al. [12] found fewer readmissions after the implementation of an occupational therapy led delirium pathway. However, in a multicentre randomised clinical trial of adults older than 75 years undergoing elective major surgery, Alvarez et al. [13] found that intensive postoperative occupational therapy did not significantly change delirium outcomes compared with standard care for people with persistent delirium (13% vs. 6%, *p* = 0.13) or subsyndromal symptoms (49% vs. 52%, *p* = 0.4) compared to controls. These studies underscore the promise of environmental modifications in hospital environments, yet their specific contributions to multidisciplinary care remain unclear. Rigorous evaluation is lacking, with few studies including comparison groups to robustly assess the efficacy of these interventions.

This study evaluated a multidimensional occupational therapy environmental checklist's impact on functional and service outcomes for hospitalised delirium patients compared to standard care. ‘Multidimensional’ refers to its five domains (orientation, sensory optimisation, identity and belonging, daily routine and environmental safety). The research question was: How do length of stay, adverse events and functional outcomes differ between intervention and standard care groups?

## 2 Methods

This study employed a quasi‐experimental design, collecting pre and post intervention data from patients receiving and not receiving the checklist intervention. Ethics approval was obtained via the health service's negligible risk (QA2022.30_85975 11/07/2022) and low risk (HREC/22/WH/8732007/09/2022) pathways and ratified by the partner university.

### 2.1 Study Setting

The study was conducted on the Geriatric Evaluation and Management (GEM) ward of a metropolitan hospital in Melbourne, Australia. The research team comprised four clinician researchers with 5–25 years of experience working with older people with delirium, and a final‐year occupational therapy student completing her Honours degree. Two of the clinician researchers (Neale, Hitch) have led previous research about the role of occupational therapy in delirium management.

### 2.2 Intervention

The Checklist aimed to create a delirium‐friendly environment for older adults admitted to hospital. It was developed based on; expertise and Occupational Therapy delirium research at Western Health and refined through clinical consultation, drawing on evidence that multicomponent non‐pharmacological interventions (particularly orientation supports, sensory correction, personal items, mobility and activity provision) reduce delirium incidence and persistence [1, 5, 7, 14, 15]. Its theoretical basis, development process and rationale for each item are detailed in File S1—Section 1.

The checklist was completed by an occupational therapist once at the point of delirium identification, with daily follow‐up checks conducted by allied health assistants (AHAs) to maintain fidelity to environmental modifications. These procedures were sustained across bed moves and used in both single and shared rooms. A detailed, Template for Intervention Description and Replication (TIDieR)‐aligned description of the intervention processes, fidelity monitoring, materials, and implementation workflow is provided in File S1—Section 2.

Implemented by occupational therapists and AHAs with multidisciplinary team support, the Checklist included interventions across multiple domains (Table 1). Training was conducted quarterly over the 12‐month period of this study, and weekly reminders and reinforcement were provided during patient Journey Board (discharge planning meetings). This training included information about delirium as a condition, the impact of delirium on patient outcomes, delirium assessment, the environmental strategies in the Checklist and multidisciplinary roles in supporting Checklist implementation. Following referral to occupational therapy, AHAs actively assessed the patient's environment using the Checklist and implemented environmental modifications as an intervention, reinstating items that had been altered or removed since the previous session.

**TABLE 1:** Delirium environmental checklist: Components and purpose.

Domain	Checklist items	Purpose/function
Orientation	Room clock displaying correct time, day, date, month and year	Constant temporal orientation, reducing misperceptions and anxiety Supports normal circadian cycles
	Bedside whiteboard updated with name, location, date and staff names	Reinforces orientation to person and place Supports communication with rotating staff Reduces confusion about roles
	Blinds up during the day and down at night	Provides natural light cues for day/night distinction Supports circadian rhythm and sleep–wake regulation
	Lights on if room is dark during the day	Reduces shadows and misperceptions Facilitates safe mobility and engagement with surroundings
	Toilet labelled with clear signage	Assists wayfinding Reduces anxiety and incontinence‐related agitation Supports independence in toileting
	Patient verbally orientated to room (i.e., location of toilet, bathroom, belongings, call bell)	Reinforces where things are and how to call for help Reduces fear and distress on waking or after staff changes
	Patient wearing their own day and night clothes	Supports personal identity, dignity and autonomy Distinguishes daytime from night Reduces ‘sick role’ passivity
Objects	Glasses, working hearing aids and mobility aids within reach	Optimises sensory input and safe mobility Reduces misinterpretation and withdrawal due to sensory loss
	Water jug and cup on tray table (with diet/fluid restrictions checked)	Supports hydration and comfort Dehydration is a modifiable delirium risk factor
	Phone and call bell within reach	Enables help‐seeking and connection with family Reduces unsafe bed exits and frustration
Identity	Personal photos, blankets and other familiar items	Provide reassurance and emotional grounding Prompts conversation Support sense of self and relationships
	Sunflower Tool (patient facts poster) commenced and displayed	Provides succinct information about past occupations, interests, preferences, and important people Supports personalised conversation and reassurance
Routine	Personal timetable displayed with daily routine	Makes the day predictable Anchors therapy, mealtimes, and preferred activities
	OT AHA referral made to support daily routine (e.g., shower/dress retraining, favourite TV)	Ensures consistent delivery of routine based and activity interventions Integrates checklist into occupational therapy/allied health assistant workflows
	Family/friends/carers encouraged to visit	Supports belonging, provides orientation and comfort Reinforces personal routines and identity
	Engagement pack provided (e.g., crosswords, books, knitting, colouring)	Offers simple, meaningful cognitive and leisure activities to reduce boredom and agitation
	Patient sits out of bed to eat meals or receives assistance to eat (if appropriate)	Promotes mobilisation, functional independence and safe nutrition Reduces deconditioning and delirium severity
Environment	Room is not cluttered—no clothing or trip hazards and unnecessary items removed	Reduces fall risk and sensory overload Supports safe mobility and calmer environment
	Delirium‐friendly environment sign on patient door	Signals to staff that delirium‐sensitive care is required Prompts use of environmental and communication strategies
Implementation	Referral made to OT AHA for weekday Delirium Patient Room Checking	Ensures ongoing daily review of environmental items, sustaining intervention fidelity beyond initial OT visit

### 2.3 Participants and Recruitment

Data were collected in two phases via electronic medical record (eMR) audits to evaluate patient outcomes. Consecutive purposive sampling identified eMR records for patients admitted to the GEM ward. Phase 1 involved a retrospective audit of electronic medical records from June 2021 to June 2022, while Phase 2 comprised a retrospective audit of patients who received the intervention between August 2022 and August 2023. This approach enabled the capture of practice prior to and after Checklist implementation into routine clinical practice, and prospective recruitment or randomisation was not feasible within organisational and ethical constraints.

Phase 1 inclusion criteria were a delirium diagnosis (medical or 4AT score of ≥ 4) on admission to GEM prior to checklist implementation and aged over 18 years. Phase 2 criteria were a delirium diagnosis on admission to GEM, aged over 18 years and exposure to the checklist. Although the checklist was developed for use with older people, the inclusion criterion of age over 18 years reflected the ward admission profile, which occasionally included younger adults with delirium. Including younger patients with documented delirium diagnosis ensured complete case capture.

The 4AT assessment [16] screens for delirium or cognitive impairment and has been validated for use with hospitalised older people [17], with a score of 4 or above indicating possible delirium. However, the results of these tests are rarely recorded in eMR records and most delirium diagnoses are made via clinical observation by medical staff. The issue of inconsistent recording of delirium diagnoses is also often reported in international studies [18, 19].

The sample size was determined by the total number of eligible patients admitted during the study, which ensured complete case capture for both phases. This approach provided a naturalistic and comprehensive dataset for comparing outcomes before and after Checklist implementation, which maximised external validity [20].

### 2.4 Procedure and Data Collection

All clinical information (delirium diagnosis, 4AT scores and adverse events) was extracted directly from the eMR with no separate paper medical records utilised. Sociodemographic variables extracted included age, gender, ethnicity, reason for emergency department presentation, reason for ward admission, comorbidities and delirium resolution at discharge. Data extracted from eMRs focussed on patient and service outcomes, including length of stay, pressure sore incidence, number of falls and Functional Independence Measure (FIM) [21] scores (motor, cognitive, and total) at both admission and discharge. The FIM assessments were completed routinely by trained and credentialled allied health clinicians as part of usual GEM ward practice. Pre‐ and post‐FIM scores were completed by the treating clinical team consistent with standard care, with no knowledge of whether the patient was included in this study.

Delirium diagnosis was sourced from the electronic medical record (eMR), including any documented 4AT scores when available. Adverse events were captured as the number of documented incidents in the RiskMan platform [22], including falls, pressure injuries, Code Grey (unarmed threats) and Code Black (armed threats). The research team extracted data into a secure RedCAP platform [23] for analysis and storage. Data extraction from the eMR and RiskMan systems was completed by EM and SN and cross‐checked by DH to ensure accuracy. Bias was therefore managed by using consecutive sampling across defined time periods, applying identical inclusion criteria to both groups, using routinely collected data from clinicians blind to study inclusion, and undertaking data extraction validation before analysis.

### 2.5 Data Analysis

Descriptive statistics, including frequencies, mean and standard deviation, were calculated using SPSS Statistics 28 to summarise demographic characteristics (age, gender, ethnicity, reason for admission, delirium diagnosis method and delirium resolution at discharge) and clinical outcomes. Independent *t*‐tests assessed differences in continuous variables (e.g., age, length of stay, FIM scores) between groups, while *χ* ^2^ tests evaluated categorical variables (e.g., gender, ethnicity, adverse events, discharge destination). A significance threshold of *p* < 0.05 was applied.

## 3 Results

Data were extracted from 50 eMR records prior to Checklist implementation and 50 eMR records following Checklist implementation.

### 3.1 Sample Demographics

Patient age did not differ significantly between comparison records (M 80.9, SD 7.5, Range 63–99) and intervention records (M 81.6, SD 8.3, Range 56–96), *t* = −0.49, *p* = 0.31. There were no significant differences between the groups for gender, ethnicity, method of delirium diagnosis and delirium resolution at discharge (Table 2).

**TABLE 2:** Demographic characteristics.

	Comparison group *n* (%)	Intervention group *n* (%)	*χ* ^2^, *p*
Gender			
Male	20 (40)	28 (56)	2.56, 0.11
Female	30 (60)	22 (44)	
Ethnicity			
Australian born and English speaking	13 (26)	17 (34)	1.64, 0.44
Overseas born and English speaking	14 (28)	9 (18)	
Overseas born and non‐English speaking	23 (46)	24 (48)	
Reason for admission^a^			
Delirium	15 (30)	10 (20)	20.38, < 0.001*
Functional goal achievement and discharge planning (21)	21 (42)	11 (22)	
Medical reasons	31 (62)	17 (34)	
Fractures or other musculoskeletal issues	7 (14)	2 (4)	
Neurological issues	5 (10)	9 (18)	
Cardiovascular issues	2 (4)	2 (4)	
Carer‐related concerns	3 (6)	0 (0)	
Delirium diagnosis			
4AT score prior to admission	4 (8)	2 (4)	1.81, 0.40
4AT during admission	5 (10)	8 (16)	
Medically diagnosed	41 (82)	40 (80)	
Delirium resolved at discharge			
Yes	9 (18)	20 (40)	6.26, 0.10
No	26 (52)	21 (42)	
Deceased	4 (8)	2 (4)	
Not documented	11 (22)	7 (14)	

^a^ More than one reason for admission was specified for most patients.

* Indicates significance.

### 3.2 Clinical Outcomes

As shown in Table 3, when comparing the number of patients experiencing any adverse event, there was no significant difference between the comparison and intervention groups (*χ* ^2^ = 1.48, *p* = 0.22). However, there was a decrease of 27% in the total number of adverse events (*n* = 27) in the intervention group in comparison with the comparison group (*n* = 37). There was no significant difference between the groups for discharge destination when comparing home, residential aged care and other destinations (*χ* ^2^ = 0.80, *p* = 0.67). There was also no significant difference for length of stay between the comparison group (M 28.2 days, SD 36.2 days) and the intervention group (M 29.36, SD 18.1), *t* = −0.20, *p* = 0.84.

**TABLE 3:** Summary of clinical outcomes.

	Comparison group		Intervention group		*χ* ^2^, *p*
	Patients *n* (%)	Incidents *n* (%)	Patients *n* (%)	Incidents *n* (%)	
Adverse events					
Any adverse events (≥ 1)	24 (48)		18 (36)		1.48, 0.22
Pressure injuries	7 (14)	7 (14)	2 (4)	2 (4)	
Inpatient falls	13 (26)	21 (42)	12 (24)	15 (30)	
Planned Code Grey	3 (6)	7 (14)	1 (2)	3 (6)	
Unplanned Code Grey	2 (4)	2 (4)	3 (6)	3 (6)	
Code Black	0 (0)	0 (0)	0 (0)	0 (0)	
Other adverse events	12 (24)	16 (32)	9 (18)	13 (26)	
Discharge destination					
Home	24 (48)		27 (54)		0.80, 0.67
Other hospital ward	5 (10)		4 (8)		
Residential aged care	16 (32)		17 (34)		
Deceased	4 (8)		2 (4)		

*Note:* Code Black = Armed aggressive behaviour, Code Grey = Unarmed aggressive behaviour.

### 3.3 Functional Independence Measure Scores

**FIGURE 1:**
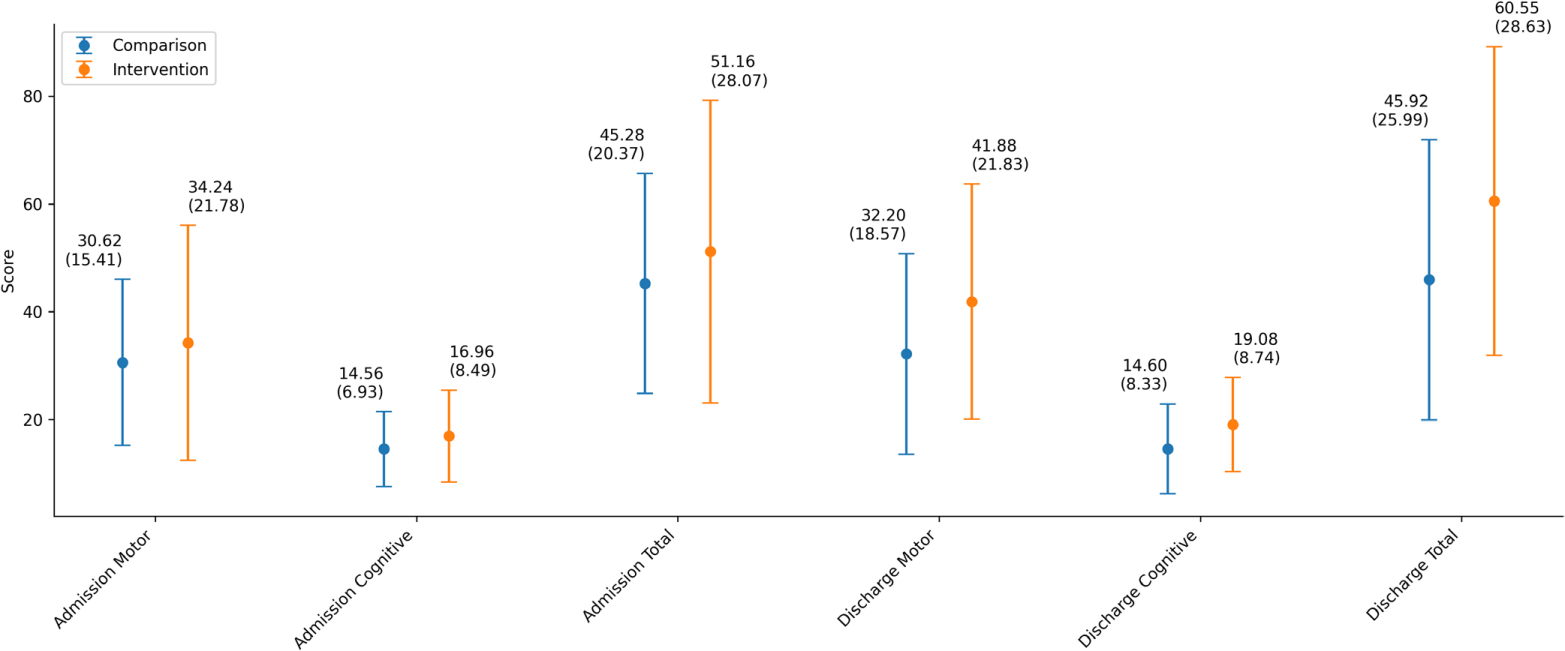
Functional independence measure scores for comparison and intervention group at admission and discharge.

As shown below in Figure 1, the intervention group had higher mean motor, cognitive and total FIM scores than the comparison group at both admission and discharge.

There were no significant differences in motor, cognitive or total FIM scores between the group at admission (Table 4). However, the intervention group achieved significantly higher motor, cognitive and total FIM scores by the time they were discharged.

**TABLE 4:** Summary of FIM scores.

	Comparison group mean (SD)	Intervention group mean (SD)	*t* score, *p*
Admission			
Motor	30.62 (15.41)	34.24 (21.78)	−0.96, 0.34
Cognitive	14.56 (6.93)	16.96 (8.49)	−1.55, 0.12
Total	45.28 (20.37)	51.61 (28.07)	−1.62, 0.11
Discharge			
Motor	32.20 (18.57)	41.88 (21.83)	−2.38, 0.02*
Cognitive	14.60 (8.33)	19.08 (8.74)	−2.62, 0.01*
Total	45.92 (25.99)	60.55 (28.63)	−3.24, < 0.001*

* Indicates significance.

## 4 Discussion

While there is growing and promising evidence to support targeted environmental interventions in hospital wards, their implementation and effectiveness remain an underexplored area in delirium management research. The findings of this study indicated the capacity of the multidimensional occupational therapy Checklist to improve functional outcomes in older adults with delirium. Implementing the Checklist resulted in important improvements for both people with delirium via significantly enhanced function at discharge and the organisation through fewer total adverse events.

The Checklist aligns closely with the national Delirium Clinical Care Standard [2], by operationalising recommended nonpharmacological strategies, actively involving families, enabling person‐centred practices and supporting the general principles of care. The findings of this study provide some evidence that implementing care guidelines that target the environment improves outcomes. Therefore, the Checklist offers a practical tool to help services implement and locally monitor adherence to these evidence‐based practices. For example, items from the orientation and sensory domains (e.g., ensuring visible clocks, calendars and adequate lighting) illustrated how the Checklist provided a structured, replicable process for modifying the care environment. Although this study focused on those diagnosed with delirium, the checklist components also map directly onto guideline‐recommended risk modifications, suggesting potential applicability as a prevention strategy that warrants prospective evaluation [6, 7].

This study complements existing evidence about the potential of environmental modifications for supporting functional recovery for older adults with delirium [5, 6]. The impacts of delirium on function can persist for months after hospitalisation [24] and are less amenable to improvement than the functional impacts of other conditions such as depression [25]. Optimising function during hospital admission through targeted environmental strategies, such as the Checklist, may therefore be a protective strategy for reducing long‐term functional decline for this vulnerable population. These findings align with previous occupational therapy‐led delirium pathways and environmental programs, which used orientation boards, structured care plans and activity‐based strategies to modify the ward environment and support recovery [10, 11, 12].

However, the lack of significant reductions in length of stay or the number of patients experiencing adverse events reflects the complex clinical presentations and contexts of delirium management. Advanced age and lower functional status are themselves risk factors for delirium [26], and other confounding variables such as falls and co‐morbidities make it challenging to isolate the specific impact of the Checklist. Future research could address these confounders by employing stratified analyses to examine the Checklist's effects across subgroups defined by age, functional status and fall history, or use propensity score matching to control for comorbidities in larger, multi‐site studies.

The Checklist's structured, occupational therapy‐led approach offers a replicable, low‐cost framework for assessing and modifying ward environments, with potential for scaling across diverse settings to enhance delirium management for older adults. Its simplicity and affordability also support its accessibility for resource‐constrained hospitals in the Australasian context, amidst rising demand from ageing populations [27]. However, participation in non‐pharmacological delirium interventions can be thwarted by poor symptom control and inflexible hospital routines and practices [28]. To maximise the Checklist's future use, hospitals will need to integrate it with symptom management protocols and be willing to adopt more flexible ward routines, both of which will support broader adoption and sustained impact.

### 4.1 Strengths and Limitations

The inclusion of a comparison group enhanced the rigour of this study, as the development of the Checklist was enhanced by experienced occupational therapists familiar with the current evidence base. The retrospective design meant that groups could not be matched on baseline characteristics and unmeasured confounding is possible. FIM assessments were completed by treating clinicians as part of routine care rather than blinded assessors, which may also introduce assessment bias. The eMR data documentation quality varied significantly, and some delirium related variables were incompletely recorded. These limitations mean causality cannot be established, but the findings still provide useful real‐world evidence of the checklist's impact in routine practice. Occupational therapist and AHA time spent implementing the checklist were not recorded, however, and could have had a confounding effect on patient outcomes.

The focus on a GEM ward enabled the recruitment of a representative group of older adults in a setting with high delirium prevalence. However, the small sample size limits the ability to detect significant differences in outcomes such as length of stay, and the generalisability of these findings. The single‐site design also means these findings may not be relevant to other settings, or contextual factors such as staffing levels or ward layout.

## 5 Conclusions

This study evaluated the impact of a multidimensional occupational therapy hospital Environmental Checklist on functional and service outcomes for older people with delirium patients compared to standard care. It demonstrated the Checklist's potential to enhance recovery, resulting in significantly higher functional scores at discharge and reduced total adverse events in the intervention group. Although length of stay and overall adverse event rates were not significantly different following Checklist implementation, the intervention group showed greater functional gains and fewer patients experiencing any adverse event. These results provide preliminary evidence that the Checklist may support improved patient outcomes through targeted environmental modifications, particularly for older adults vulnerable to delirium‐related decline, while organisational impacts such as length of stay require confirmation in larger studies.

Future research should prioritise larger, multi‐site studies with extended follow‐up periods to confirm the Checklist's efficacy and generalisability across diverse healthcare settings, addressing the current single‐site limitation. Incorporating retrospective comparison groups or advanced statistical methods, such as propensity score matching, would strengthen the evidence base for this intervention. Including patient and family perspectives will be crucial to ensure the Checklist meets their needs and could potentially enhance its impact through co‐designed modifications and improvements. The cultural validity of the Checklist should also be explored and developed to better reflect the multicultural nature of Australasian communities. From a practical point of view, the Checklist will need to be integrated into routine care through staff training and implementation support and may require tailoring for specific ward contexts.

Overall, this study contributes to knowledge and practice related to multidimensional interventions for delirium management by providing evidence of the value of structured environmental interventions. It also provides further evidence of the key, but often overlooked, role that occupational therapy could play in effective delirium management during hospital admissions.

## Funding

The authors have nothing to report.

## Ethics Statement

This study was approved by the Western Health Human Research Ethics Committee under negligible risk (QA2022.30_85975) and low risk (HREC/22/WH/87320) pathways and was registered with Deakin University's ethics committee.

## Conflicts of Interest

The authors declare no conflicts of interest.

## Supporting information


**File S1:** ajag70165‐sup‐0001‐supinfo.docx.

## Data Availability

The data that support the findings of this study are available from the corresponding author upon reasonable request.
