# Supplementary material for: Evaluation of a Multidimensional Occupational Therapy Environmental Checklist for People Experiencing Delirium During Hospital Admission: A Quasi‐Experimental Study
Source: Australas J Ageing. 2026 Apr 24;45:e70165. doi: 10.1111/ajag.70165 (PMC13108554; doi:10.1111/ajag.70165)
Supplement: Supplementary file 1 — File S1: ajag70165‐sup‐0001‐supinfo.docx. [file AJAG-45-0-s001.docx]

**Supplementary File**

**Section 1**

**Development of the Delirium Environmental Checklist**

The Delirium Environmental Checklist was developed at Western Health, Melbourne, Australia as an occupational therapy-led response to persistent gaps in delirium care, particularly around the optimisation of hospital environments for older adults at risk of, or experiencing, delirium. Its development drew together several strands of ongoing work by the occupational therapy department at Western Health including previous Honours research projects, a review of the growing international evidence for multicomponent delirium interventions, reflection on occupational therapy theory, particularly the Pan Occupational Paradigm (POP),^1^ and iterative feedback from clinicians working in acute and subacute settings.

**1.1 Contributing knowledge and practice**

*1.1.1 University/Health Service collaborative research*

Early work by Western Health occupational therapists highlighted that hospital environmental factors contributing to delirium were rarely addressed in a systematic way. Five mixed Honours research studies examined delirium care, staff knowledge, carer needs and environmental modification strategies within the health service,^2-5^ along with knowledge, skills and attitudes across the Australian occupational therapy workforce.^6^ These projects consistently identified that orientation cues were inconsistent, sensory aids such as glasses and hearing aids were frequently missing or out of reach, personal items were seldom incorporated into care, and there was no standardised process for modifying the environment around people with delirium. At the same time, staff expressed a desire for clearer guidance and practical prompts to support delirium care in busy wards. Together, these findings indicated a need for a structured, occupational therapy led environmental tool to make evidence-based strategies visible, repeatable and shareable across the multidisciplinary team.

Two of these studies specifically evaluated and informed the ongoing development of the Checklist. In 2022,Western Health^2^ evaluated whether the Checklist could improve functional and service outcomes on a Geriatric Evaluation and Management (GEM) ward at Sunshine Hospital. The study used a two-phase design: a retrospective audit of electronic medical records to describe outcomes under standard care, followed by a prospective audit after checklist implementation, alongside surveys and focus groups with GEM multidisciplinary staff to explore their perceptions of the intervention and its implementation. Quantitative findings suggested that patients exposed to the checklist experienced greater functional gains on the Functional Independence Measure (FIM) and fewer adverse events than those receiving standard care, while qualitative data indicated that occupational therapists and allied health assistants saw the Checklist as feasible and valuable but time consuming, highlighted the importance of MDT and family contributions, described the limitations of the physical ward environment, and recommended clearer wording on some items and the promotion of shared ownership of delirium-friendly care. These findings directly shaped the current Checklist by confirming the relevance of specific components (for example, orientation cues, personal items, sitting out of bed, Sunflower tool), prompting clearer grouping and wording of items, embedding its use in routine occupational therapy/allied health assistant workflows, and strengthening the rationale for this occupational therapy led but multidisciplinary team supported intervention. Feedback from this study also led to the solving of small practical issues, such as ensuring enough clocks and activity kits were available on the ward. Additional allied health assistant hours were negotiated to support daily Checklist work without displacing other essential tasks.

In 2024, Sfara^4^ examined how workable, acceptable and useful the Checklist was when embedded into routine practice on a ward which admitted both aged care and acute patients at Sunshine Hospital. Using a two-phase mixed-methods design, she first audited electronic medical records and surveyed multidisciplinary team members to describe baseline outcomes and existing delirium strategies. Sfara then repeated the audit after Checklist implementation alongside a second survey, plus focus groups and interviews exploring acceptability, demand, implementation, practicality and adaptation. The findings confirmed the Checklist could be integrated into existing occupational therapy/allied health assistant workflows in a different setting. It was generally viewed by staff as acceptable, appropriate and feasible, and was associated with improved functional outcomes and fewer adverse events. However, the key barriers on this ward included time pressures, inconsistent delirium documentation, and variable multidisciplinary team and family involvement. These findings reinforced the central role of allied health assistants in daily environmental checks, which was reinforced within Checklist procedures. It also supported a plan to formalise Checklist documentation within the electronic data base, helped prioritise the most consistently used and effective items (for example, orientation cues, sensory aids, access to water, toilet signage and decluttered spaces), and underscored the need for ongoing multidisciplinary education and shared ownership of ‘delirium-friendly’ environments, rather than viewing the Checklist as an occupational therapy specific intervention.

*1.1.2 Iterative review of delirium literature*

The international delirium literature continues to provide a strong evidentiary foundation for the content of the Checklist. Multicomponent interventions aimed at delirium prevention and management, like those reviewed by Hshieh et al^7^ and Ludolph et al,^8^ demonstrate that interventions combining orientation, sleep–wake regulation, mobilisation, sensory correction, hydration, nutrition and cognitive stimulation can reduce the incidence of delirium and shorten hospital stays compared to usual care. Subsequent work has reinforced that interventions addressing these essential needs are most effective when they target multiple delirium risk factors simultaneously.^9, 10^ In parallel, studies of specific delirium programs, including the ‘Eat Walk Engage’ model and similar ward-based initiatives, highlighted the importance of environmental cues, activity provision, and sustained staff engagement, often supported by allied health assistants to maintain adherence.^11, 12^

This evidence also supported individual components that now appear in the Checklist. Martínez and colleagues^13^ reported that a family-delivered environmental orientation interventions, including use of clocks, calendars and familiar objects in the patient’s vicinity, significantly reduced the incidence of delirium among older inpatients (relative risk 0.41, 95% CI 0.19–0.92; *p* = 0.03). Mudge et al^12^ demonstrated that multicomponent ward level interventions can lower hospital associated delirium while also drawing attention to the role of allied health assistants in sustaining environmental and activity-based strategies. Other studies contributed evidence for orientation boards, appropriate lighting, minimising bed moves, and the provision of meaningful activities and personal items as ways to support identity, comfort and engagement in people with delirium.^14-18^ Together, this literature offered a range of proven or promising component options that were translated into the Checklist.

*1.1.3 Reflection on occupational therapy foundations*

The theoretical framing for the Checklist came from the Pan Occupational Paradigm (POP), which was adopted as the overarching conceptual lens for the broader delirium research program at Western Health^1, 19^ The POP extends Wilcock’s original ‘doing, being, becoming, and belonging’ framework^20^ by adding a fifth dimension—resources and opportunities—and emphasises the flexible, adaptive relationship between occupation and health. Within this paradigm, older people in hospital are viewed as occupational beings whose wellbeing is shaped by their opportunities to engage in meaningful activities, maintain their identity, and sustain connections with others, even in the context of acute illness.

The development team have aligned Checklist content with these POP dimensions to ensure the maintenance of an occupational perspective of delirium. Items related to orientation and sensory aids are understood as supporting ‘being’ (the person’s sense of self and capacity to interpret their surroundings) and ‘resources and opportunities’ (the environmental conditions that enable or constrain engagement). Prompts about personal clothing, photos, and the Sunflower ‘about me’ tool are framed around ‘belonging’ and identity, reinforcing the person’s social roles and relationships. Encouraging patients to sit out of bed for meals, participate in simple activities, and follow a personal timetable reflect ‘doing’ and ‘becoming’, supporting functional recovery and a sense of progress. By grounding the Checklist in the POP, the team ensured that environmental modification would not be reduced to a series of isolated tasks but instead reflect a coherent occupational perspective on delirium care.

**1.2 Process of development**

With the evidence base and theoretical framework established, senior occupational therapists at Western Health drafted the initial version of the Checklist. This initial version drew directly on the gaps and opportunities identified in the Honours studies, as well as everyday clinical observations from acute and sub-acute wards. Items were chosen to reflect practical, observable aspects of the bedside environment that could be modified without requiring additional medical orders. Examples included whether clocks and calendars were visible and correct, whether whiteboards accurately displayed the date and staff names, whether glasses, hearing aids, mobility aids and call bells were within reach, whether the patient was dressed in their own day clothes, and whether personal photos or objects were present. The draft also included prompts about activity resources (such as simple engagement packs) and seating arrangements for meals.

This draft Checklist was then refined through a process of multidisciplinary consultation. Occupational therapists, allied health assistants, nurse unit managers, and medical staff were invited to review the content and discuss its feasibility within existing workflows. These discussions highlighted the need for clear division of roles, particularly which tasks should be led by occupational therapists, which were appropriate for allied health assistants, and where nursing input was essential. This led to adjustments in wording and sequencing to better reflect real world work patterns on the wards. For example, occupational therapists were identified as best placed to complete the initial Checklist at the point of delirium screening or diagnosis, drawing on their training in cognitive assessment and functional evaluation.^6, 21^ Allied health assistants were recognised as key to delivering daily environmental checks and simple modifications under occupational therapist supervision, mirroring the successful deployment of allied health support workers in other delirium programs.^12, 17^

To support consistent use of the Checklist, a suite of education resources was also developed for different staff groups. Education sessions for occupational therapists introduced the evidence underpinning the Checklist, clarified their screening and leadership role, and provided examples of how environmental changes could be integrated with other occupational therapy interventions such as functional training and discharge planning. For allied health assistants, training focused on recognising delirium, understanding the purpose of each Checklist item, and safely implementing environmental changes such as decluttering rooms, adjusting lighting and blinds, and ensuring items were within reach. Separate education for the broader multidisciplinary team emphasised that delirium management was a shared responsibility, and that the Checklist could be used to prompt communication and collaborative problem solving around environmental needs. These education packages were delivered via in-service sessions and PowerPoint presentations and reinforced during routine meetings such as journey boards and ward business meetings.

The development team also worked with health information staff to create an electronic medical record (eMR) template for documenting Checklist completion and actions. This template allowed staff to record, for each item, whether it was already in place, absent, or absent but addressed through a specific intervention. It also provided space for brief narrative notes, for example indicating that a family member had been contacted to bring in clothing or hearing aids, or that environmental changes had been negotiated with nursing staff. Embedding the Checklist in the eMR ensured that environmental interventions were visible across professions and shifts and created a data source for subsequent audit and research.

**1.3 Outcome of development**

The final Delirium Environmental Checklist that emerged from this process is a 20-item tool. Each item is framed as a concrete, observable environmental feature; for example, whether a working clock is visible from the bed, whether the patient is wearing their own clothes, and whether the room is free of unnecessary equipment or trip hazards. The Checklist can record both baseline conditions and the actions taken to optimise the environment. By structuring the tool this way, the development team ensured that it could be used flexibly, as a one-off audit, as a daily intervention checklist, and as a communication tool between staff members.

In summary, the Delirium Environmental Checklist was developed through a rigorous, iterative process that integrated local research, international evidence, occupational therapy theory and multidisciplinary clinical expertise. It translates well established elements of multicomponent delirium interventions into a practical, ward friendly format grounded in occupational therapy theoretical foundations. As such, it provides a structured means for occupational therapists, allied health assistants and the wider multidisciplinary team to enact an occupational perspective on delirium management in everyday practice, and creates a platform for ongoing evaluation and refinement of environmental strategies in this area of care.

**Section 2**

**Comprehensive description of the delirium environmental checklist intervention** *(based upon the TIDieR Checklist^22^)*

**2.1 Brief name**

Delirium Environmental Checklist

**2.2 Why (Rationale, Theory, or Goal)**

The Checklist is grounded in evidence that multicomponent non-pharmacological delirium interventions reduce incidence, duration and severity of delirium.

The theoretical underpinning draws on the Pan Occupational Paradigm (POP),^1^ emphasising ‘doing, being, belonging, becoming, and resources/opportunities’ as essential structures for supporting occupational engagement, identity and recovery. The Checklist operationalises these principles through environmental adjustments that enhance orientation, sensory access, identity cues, mobility and engagement opportunities.

Its overall goal is to create a ‘delirium friendly’ environment that supports functional recovery, reduces adverse events, and promotes therapeutic engagement between patients, families, and staff.

**2.3 What: Materials**

*2.3.1 Physical materials*

- Delirium Environmental Checklist (20-item audit tool)
- Occupational therapist/allied health assistant workflow documents describing screening, initial audit, and daily follow-up
- Delirium engagement/activity packs (crosswords, colouring, magazines, simple cognitive tasks)
- Orientation resources including clocks, calendars, whiteboard, signage for toilets and bathrooms
- Personal item prompts including clothing, glasses, hearing aids, familiar objects, ‘Sunflower Tool’/‘About Me’ poster
- Decluttering resources including bags/containers for equipment, storage spaces

*2.3.2 Informational materials*

- Printed education handouts and PowerPoint slides (occupational therapists, allied health assistants, and multidisciplinary staff versions) addressing role clarification, rationale, examples of environmental optimisation
- eMR documentation template with three response categories: Present; Absent; Absent and Addressed
- Occupational therapy delirium screening guidance (including 4AT thresholds, inclusion/exclusion criteria)
- Allied health assistants daily scheduled tasks sheet describing steps for daily room checks

All materials are stored internally within Western Health (hardcopy on ward; electronic versions on staff drive and via SharePoint). The structured eMR template is available within the organisation’s electronic medical record system.

**2.4 What: Procedures**

*2.4.1 Initial OT-led implementation*

1. Screening: Occupational therapist identifies patient with delirium or at high risk (via medical diagnosis or 4AT ≥4)
2. Complete initial Checklist at bedside, marking each item as Present, Absent, or Absent and Addressed
3. Intervene immediately by
   - Updating whiteboards; adjusting lighting/blinds; ensuring clocks/calendars are correct
   - Repositioning or locating glasses/hearing aid
   - Decluttering room and removing hazards.
   - Requesting family to bring personal clothes, photos, or familiar objects
   - Providing an activity/engagement pack

*2.4.2 Allied health assistants daily follow-up procedures*

1. Daily room check (Mon–Fri) using the same Checklist
2. Implement simple environmental modifications (orientation aids, item placement, decluttering)
3. Document actions in the eMR template
4. Escalate issues to occupational therapist (e.g. high falls risk, missing sensory aids, persistent agitation)
5. Liaise with nursing staff regarding seating for meals, mobility, and toileting access

*2.4.3 Multidisciplinary team engagement*

- Nurses contribute to maintaining whiteboards, toileting signage, and lighting routines
- Families are encouraged to provide personal items and maintain routine familiarity
- Medical staff informed of changes through eMR entries and journey boards

**2.5 Who Provides the intervention**

*2.5.1 Occupational therapists*

- Role: Screening, initial Checklist completion, complex interventions, supervision of allied health assistants
- Training: Education session on delirium evidence, Pan Occupational Paradigm framework, and tool use (via occupational therapist education slides)
- Expertise: Cognitive assessment, functional mobility, environmental modification

*2.5.2 Allied health assistants*

- Role: Daily room checks and environmental interventions; documentation in eMR
- Training: Dedicated education session covering delirium recognition, checklist steps and safety considerations (via allied health assistants PowerPoint)

*2.5.3 Nursing staff*

- Role: Support orientation cues and daily routine; update whiteboards; assist with decluttering
- Training: Brief multidisciplinary team education session on delirium and environmental strategies

*2.5.4 Families*

- Role: Provide clothing, personal items, photos and identity cues
- Training: Verbal explanation by occupational therapists/allied health assistants; family information sheet

**2.6. How (modes of delivery)**

- Face-to-face bedside delivery for both occupational therapist and allied health assistant components
- Documentation via eMR
- Communication with multidisciplinary team through journey boards, ward meetings and bedside handover
- Family engagement via in-person conversations, phone calls and written prompts

**2.7 Where?**

- Geriatric Evaluation and Management (GEM) ward
- Mixed ward comprising two teams (acute care and sub-acute GEM) located on one physical ward
- All sub-acute patients are seen by occupational therapists, but acute patient are only seen if referred
- Environment includes single and shared rooms, centralised nursing station, therapy areas and communal dining spaces

**2.8 When and how much?**

- Initial checklist: Completed by occupational therapist once per patient at time of identification of delirium or risk (approximately 30 minutes)
- Allied health assistants’ daily checks: Once daily (Mon–Fri), each session typically taking 5–10 minutes depending on complexity
- Duration of intervention: Continues until delirium resolves or patient is discharged.
- Occupational therapy review sessions: Frequency determined by clinical need

**2.9 Tailoring**

- Personalisation is central to the intervention. For example:
  - Family provided items vary per patient (clothing, photos, hobbies)
  - Activity pack contents are selected based on cognitive and sensory needs
  - Adjustments differ depending on mobility level, sensory impairments and ward behaviour
- The Checklist allows ‘Absent and Addressed’ actions tailored to each patient’s needs and circumstances
- Tailored decision making is informed by occupational therapy assessment and allied health assistants’ daily observation

**2.10 Modifications during study period**

- Language of some items was simplified following staff feedback (e.g. ‘items within reach’)
- Domain headings were clarified (e.g. Orientation, Belonging, Safety)
- Additional allied health assistant hours were allocated to support checklist consistency
- Activity pack content was standardised based on feasibility

**2.11 How Well (planned fidelity)?**

- Fidelity monitored through:
  - eMR documentation of each checklist item daily
  - Supervision meetings between occupational therapists and allied health assistants
  - Ward audit spreadsheets tracking delirium cases and checklist completion, periodically analysed to inform quality improvement

Planned fidelity strategies include education sessions, use of standardised checklist, clear occupational therapist/allied health assistant workflows and integration into eMR to improve visibility.

**2.12 How Well (actual fidelity)?**

- Allied health assistants completed daily checks on weekdays only, so fidelity on weekends depends on nursing uptake
- Research showed most environmental items could be addressed daily, though challenges included:
  - Access to clocks/calendars on weekends
  - Inconsistent presence of personal items early in admission
  - Variability in nursing contributions to whiteboard updates
- Staff feedback indicated the Checklist was generally feasible but time intensive, leading to it being de-prioritised during busy periods.

**References**

1. Hitch D, Pepin G and Stagnitti K. The pan occupational paradigm: development and key concepts. *Scand J Occup Ther* 2018; 25: 27-34. 20170607. DOI: 10.1080/11038128.2017.1337808.

2. McKnight E. *Do optimised hospital environments enhance outcomes for people experiencing a delirium?​*. [REDACTED], 2022.

3. Chan C. *What Knowledge and Skills do Caregivers for Older People Treated in Tertiary Healthcare Need to Engage in Delirium Management, from the Perspectives of Both Caregivers and Occupational Therapists?* (Unpublished Honours thesis). Geelong: Deakin University; 2025.

4. Sfara M. *Feasibility of an Occupational Therapy-Led Environmental Checklist for Delirium: A Mixed Methods Study.* (Unpublished Honours thesis). Geelong: Deakin University; 2024.

5. Ward E. *Understanding the Role of Occupational Therapy with People Experiencing Delirium*. (Unpublished Honours thesis). Geelong: Deakin University; 2021

6. Strecker C and Hitch D. Perceptions of current occupational therapy practice with older adults experiencing delirium. *Australasian Journal on Ageing* 2020; 40. DOI: 10.1111/ajag.12882.

7. Hshieh TT, Yue J, Oh E, et al. Effectiveness of multicomponent nonpharmacological delirium interventions: a meta-analysis. *JAMA Intern Med* 2015; 175: 512-520. DOI: 10.1001/jamainternmed.2014.7779.

8. Ludolph P, Stoffers-Winterling J, Kunzler AM, et al. Non-Pharmacologic Multicomponent Interventions Preventing Delirium in Hospitalized People. *J Am Geriatr Soc* 2020; 68: 1864-1871. 20200612. DOI: 10.1111/jgs.16565.

9. Hosie A, Siddiqi N, Featherstone I, et al. Inclusion, characteristics and outcomes of people requiring palliative care in studies of non-pharmacological interventions for delirium: A systematic review. *Palliat Med* 2019; 33: 878-899. 20190628. DOI: 10.1177/0269216319853487.

10. Kim YH, Kim NY and Ryu S. Effects of non-pharmacological interventions for preventing delirium in general ward inpatients: A systematic review & meta-analysis of randomized controlled trials. *PLoS One* 2022; 17: e0268024. 20220506. DOI: 10.1371/journal.pone.0268024.

11. Mudge AM, Maussen C, Duncan J and Denaro CP. Improving quality of delirium care in a general medical service with established interdisciplinary care: a controlled trial. *Internal Medicine Journal* 2012; 43: 270-277. DOI: <https://doi.org/10.1111/j.1445-5994.2012.02840.x>.

12. Mudge AM, McRae P, Banks M, et al. Effect of a Ward-Based Program on Hospital-Associated Complications and Length of Stay for Older Inpatients: The Cluster Randomized CHERISH Trial. *JAMA Internal Medicine* 2022; 182: 274-282. DOI: 10.1001/jamainternmed.2021.7556.

13. Martinez FT, Tobar C, Beddings CI, et al. Preventing delirium in an acute hospital using a non-pharmacological intervention. *Age Ageing* 2012; 41: 629-634. 2012/05/17. DOI: 10.1093/ageing/afs060.

14. Bauernfreund Y, Butler M, Ragavan S and Sampson EL. TIME to think about delirium: improving detection and management on the acute medical unit. *BMJ Open Qual* 2018; 7: e000200. 20180813. DOI: 10.1136/bmjoq-2017-000200.

15. Cody S, Lizarondo L, McArthur A, et al. Improving the quality of delirium practices in a large Australian tertiary hospital: An evidence implementation initiative. *Australian Journal of Advanced Nursing* 2021; 38: 3-12.

16. Pozzi C, Lanzoni A, Lucchi E, et al. Activity-based occupational therapy intervention for delirium superimposed on dementia in nursing home setting: a feasibility study. *Aging Clinical and Experimental Research* 2020; 35: 827-833. DOI: <https://doi.org/10.1007/s40520-019-01422-0>.

17. Pozzi C, Tatzer V, Alvarez E, et al. The applicability and feasibility of occupational therapy in delirium care. *European Geriatric Medicine* 2020; 11: 209-216.

18. Siddiqi N, Harrison JK, Clegg A, et al. Interventions for preventing delirium in hospitalised non-ICU patients. *Cochrane Database Syst Rev* 2016; 3: Cd005563. 20160311. DOI: 10.1002/14651858.CD005563.pub3.

19. Hitch D and Pepin G. Doing, being, becoming and belonging at the heart of occupational therapy: An analysis of theoretical ways of knowing. *Scand J Occup Ther* 2021; 28: 13-25. 20200224. DOI: 10.1080/11038128.2020.1726454.

20. Wilcock A and Hocking C. *An Occupational Perspective of Health*. 3rd ed. Thorofare, NJ: SLACK Incorporated, 2015.

21. Deemer K, Myhre B, Oviatt S, et al. Occupational therapist-guided cognitive interventions in critically ill patients: a feasibility randomized controlled trial. *Can J Anaesth* 2023; 70: 139-150. 20221116. DOI: 10.1007/s12630-022-02351-9.

22. Hoffmann TC, Glasziou PP, Boutron I, et al. Better reporting of interventions: template for intervention description and replication (TIDieR) checklist and guide. *BMJ* 2014; 348: g1687. 2014/03/13. DOI: 10.1136/bmj.g1687.
